# Is the human face a biomarker of health? – A scoping review

**DOI:** 10.1371/journal.pone.0318138

**Published:** 2025-08-25

**Authors:** Weronika M. Obrochta, Magdalena Klimek, Paula Bartecka, Katarzyna Klaś, Urszula M. Marcinkowska

**Affiliations:** 1 Department of Environmental Health, Faculty of Health Sciences, Jagiellonian University Medical College, Krakow, Poland; 2 Jagiellonian University Medical College, Doctoral School of Medical and Health Sciences, Krakow, Poland; 3 Research Ethics in Medicine Study Group (REMEDY), Jagiellonian University Medical College, Krakow, Poland; Northumbria University, UNITED KINGDOM OF GREAT BRITAIN AND NORTHERN IRELAND

## Abstract

There is a widespread assumption that facial features could act as a biomarker of human developmental stability and thus provide an important cue to an individual’s physical and mental health status. However, research to date shows mixed support for this assumption. This is the first review that explores the association between various aspects of health and facial features, namely symmetry, averageness, and sexual dimorphism in adults. We searched electronic databases including Web of Science, MEDLINE PubMed, Scopus, and Embase. We followed the Preferred Reporting Items for Systematic reviews and Meta-Analyses extension for Scoping Reviews (PRISMA-ScR) guidelines for reporting of our results. Of the 702 screened articles, 21 were eligible for inclusion. Studies presented various relationships between facial features and cardiovascular health; immunocompetence; oxidative stress level; cortisol level; reproductive health; cognitive health, and general physical health. This review results in an inconclusive answer to the question of whether facial features can serve as honest indicators of health. The results warrant caution when utilizing facial features as a biomarker of health status and biological condition of an individual. Protocol: Open Science Framework, https://osf.io/dv9pu/.

## Introduction

### Facial features in relation to health status

Facial appearance plays a crucial role in social interactions [1]. In line with theories of sexual selection, facial features can presumably act as indicators of an individual’s age, mating success, sexual behaviour, and health [2]. Previous research has identified several facial features that may serve as indicators of an individual’s health status. These traits include, e.g., symmetry, averageness, sexual dimorphism (masculinity and femininity), adiposity, and skin colour. Sexual dimorphism, averageness, and symmetry have received extensive research attention [2]. Facial fluctuating asymmetry and averageness were suggested as morphological cues reflecting developmental stability and consequently being a proxy for an individual’s biological condition [3]. Developmental stability is defined as the capability of an organism to sustain a consistent phenotype, despite potentially disruptive genetic and environmental factors encountered during prenatal and postnatal life. It is regarded as a vital component of an individual’s survival and reproductive success [4]. A beneficial early-life environment is considered to be related to higher bilateral symmetry of facial traits, whereas pronounced asymmetries might indicate a disruption of developmental stability [5]. Sexually dimorphic features differ between the average, typical female and male phenotypes of a given species. These secondary sexual traits are being shaped under the influence of sex-typical hormones, androgens and estrogens, and were suggested to be related to one’s health and reproductive potential [6–9].

### Facial fluctuating asymmetry

Fluctuating asymmetry (FA) refers to random, small deviations from perfect bilateral symmetry, typically calculated across multiple traits. It is also regarded as an indicator of an organism’s capacity to withstand or mitigate environmental disturbances during prenatal development [10,11]. For individuals manifesting minimal deviations from ideal symmetry, low FA indicates potential developmental stability and high biological quality [12]. However, in the up-to-date studies results for FA as a marker of health are mixed and inconclusive [13–15].

### Facial averageness

Facial averageness, next to symmetry, is thought to reflect an individual’s ability to withstand the negative impacts of genetic and environmental stressors during development [2,16,17]. Facial averageness is also often associated with attractiveness [18–20]. Furthermore, it was found that individuals with average traits have higher fitness (i.e., higher number of offspring). Therefore, facial averageness could also signal reproductive health [21]. On the other hand, facial distinctiveness can be considered as the opposite of averageness, and its relationship with actual health has also been examined. A negative correlation was observed between facial averageness and semen quality; however, no association was found between averageness and immune function [2]. An inverse relationship between distinctiveness and other aspects of measured health was also observed [13]. Nevertheless, similarly to asymmetry, the results of the published studies are incongruent [9,22,23].

### Facial sexual dimorphism

More masculine or more feminine facial features were previously suggested as indicators of sex hormone exposure during development [24]. More masculine facial features are manifested in a strong jaw, brow ridge, and higher facial weight-to-height ratio [25]. Facial masculinity may convey information about health through its association with testosterone [26]. Testosterone, while enhancing overall masculinity or muscle mass, can also compromise health by suppressing immune functions [22] and elevating oxidative stress levels [27]. Testosterone also plays a vital role in spermatogenesis [28], suggesting that masculinity may also be related to semen quality [2]. More feminine facial features are defined as fuller lips, bigger and rounder eyes, and a narrow chin. Facial femininity has been suggested to be linked with estrogens [29], which also play a role in enhancing immune function [30] and reproductive health, given that higher levels of estradiol are needed for a successful ovulation [31,32]. Nevertheless, the impact of sex hormones on women’s health remains a topic of debate and is possibly not as strong as in men [22].

### Perceptual and measurement approaches to facial features

Studies included in the current review were designed employing varying methodologies, assessing facial shape based on the perception of participants or based on actual facial measurement. In the Brunswik lens model [33], the former is a direct correlation, which can sometimes be accurate, but need not necessarily be so, since it can be done through the cues, i.e., cue utilisation. Judges rating the facial photographs can use, for example, averageness when they are rating health. The latter approach is framed as cue validity – there are some measurable facial features of the faces that link to health. The perceptual approach to assessments of facial features involves participants evaluating the facial appearance of presented facial stimuli [34]. Evaluators are randomly assigned to grade facial photographs on one of the chosen traits, e.g., perceived health or attractiveness, via Alternative Forced Choice or on a Likert Scale (interestingly, the results derived from either of the two methodologies are not highly correlated) [35]. For each trait, the average score for the designated face is calculated by averaging all evaluators’ ratings [2], sometimes accounting for the within and between rater agreements [36,37].

The measurement approach involves applying landmarks to the facial photographs and establishing their coordinates on a 2- or 3-dimensional grid. It is worth mentioning the importance of standardisation of photographs, i.e., employing Frankfurt horizontal plane (participant’s head position, with the camera set at his or her eye level) [38], constant lighting, and distance between the camera and participants. To standardize the location, orientation, and scale of landmarks and semi-landmarks configurations are employed, i.e., via superimposed generalised Procrustes analysis. The digitizing process is frequently carried out in the program tpsDig2 or Geomorph package [23,38], but previously other methods were also employed [39]. The landmarking can be done manually or automatically (with slight differences between the two techniques [40]).

### The aim of the scoping review

So far, no review has been conducted that would comprehensively describe the up-to-date literature on the association between facial features and health status. Our review fills this gap. It is of great importance, as frequently the connection between facial features and underlying “biological quality” is assumed and treated as a given. This stand is reinforced by the evolutionary rationale for the bases of what humans find attractive (and why attractiveness to, e.g., symmetry or sexual dimorphism would be adaptive) [41]. The research question is: What evidence exists regarding the relationship between facial features and both physical and mental health? This scoping review offers a summary of findings on the relationship between health and facial features (symmetry, averageness, and sexual dimorphism), while simultaneously stratifying the results by types of health measurements and accounting for the employed approach in facial features evaluation (perceptual or measurement).

## Materials and methods

### Protocol, registration, and reporting methods

We conducted this scoping review according to JBI (formerly Joanna Briggs Institute) methodology for scoping reviews [42] guided by a protocol registered in Open Science Framework (https://osf.io/dv9pu/). We reported this review following the Preferred Reporting Items for Systematic Reviews and Meta-Analyses extension for scoping reviews (PRISMA-ScR, see S1 Table) [43].

While this summary reports statistical tests employed in all mentioned articles, we would like to underline that readers should keep in mind also the quality of the listed studies. The review serves to describe the area of interest and was not designed to provide a quantitative answer to the research questions posed

### Search strategy

We implemented the three-step search strategy endorsed by JBI for conducting scoping reviews [42]. To build our search strategy, we conducted a series of pilot searches of the relevant databases to identify articles focused on the topic and appropriate keywords. An initial pilot search of Web of Science, MEDLINE PubMed, Cochrane Database, and Embase was conducted by the first author (WMO). The analysis of terminology within the articles allowed for the development of a full search strategy. Records obtained from the pilot search were reviewed to confirm the appropriateness of the keywords used to identify articles related to the scoping review’s research questions. Based on that, we developed the following search strategy: *“health” AND (facial AND ((symmetry OR averageness OR dimorphism) NOT (attractiveness OR palsy))) NOT children*.

The databases searched for the final analysis were MEDLINE PubMed, Web of Science, Scopus, and Embase. The final search was conducted on March 1, 2024. Two independent judges (WMO and PB) screened the reference lists of all included articles for further studies.

### Eligibility criteria

**Concept and Context.** The concept of interest focused on studies evaluating the association between health status and chosen facial features. We included studies if 1) they examined facial symmetry, averageness, or sexual dimorphism in relation to health, 2) they assessed health through analysis of biological material (i.e., blood samples, saliva samples), or medical records. We included studies with measurement and perceptual approaches to assessing facial features. We excluded articles reporting on participants’ health measured retrospectively, e.g., a survey about health state in childhood. **Participants**. We included studies involving individuals over 18 years old. We excluded studies that report results of individuals with a history of craniofacial surgeries, Bell’s palsy, lip cleft, or current or past extensive orthodontic treatment. **Types of Sources**. The scoping review considered observational and quantitative studies that describe the relationship between facial features and health. Qualitative studies, case reports, books, letters, and correspondence were excluded. **Publication date**. There were no restrictions on the publication dates of articles other than the search timing. **Language**. Only studies published in English were included in the review.

### Study selection

**Fig 1:**
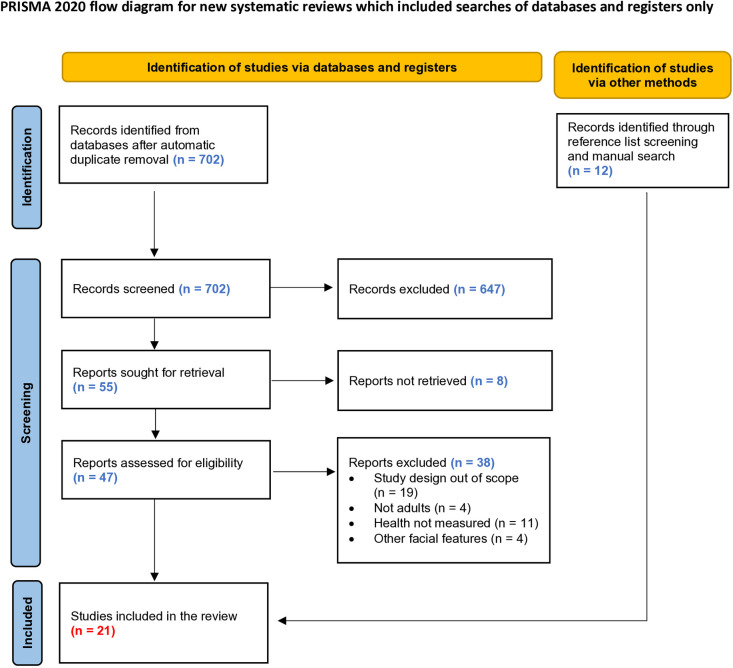
PRISMA 2020 flow diagram.

Following the search, all identified papers were collected and uploaded into EndNote 21.2.0.17387 (2023). Duplicates were removed. Titles and abstracts were screened by two independent reviewers (WMO and PB) for assessment against the eligibility criteria. The full texts of selected articles were assessed in detail against the eligibility criteria by two independent reviewers (WMO and PB), see Fig. 1 for more details. Data was screened by two independent reviewers (WMO and PB) using a data screening tool – Rayyan [44]. The reviewers (UMM and MK) resolved any disagreements that arose through discussion or with additional participation.

### Data extraction

We extracted details about the participants, concept, context, study methods, and key findings relevant to the review questions. Two reviewers (WMO and PB) independently gathered the data from each study and extracted information on the relationship between facial features and health status, and employed methodology.

### Quality assessment

Quality assessment was not conducted, as per the JBI guidance on scoping reviews [42]. The methodological quality of the included observational studies was assessed using the selected items from the Newcastle-Ottawa Scale [45], which evaluates studies based on the selection of participants, comparability of study groups, and ascertainment of exposure or outcomes.

### Data synthesis and analysis

Public health studies are often complex, as measuring human health holistically is virtually impossible. They investigate diverse populations and are characterised by heterogeneous endpoints or varied methodological approaches. As a result of the complexity, significant heterogeneity in the analysed research was expected.

We used tables for synthesis, and descriptive summaries were utilised for reporting on the article characteristics. Two reviewers (WMO and PB) independently and then collaboratively (with UMM and MK) prepared the results’ tables. We compared similarities based on the facial features studied and types of health measurements. Due to the multiple approaches to health measurements, for the sake of scientific clarity we developed categorisation guide to segregate results as follows: 1) cardiovascular health; 2) immunocompetence; 3) oxidative stress level; 4) cortisol level; 5) reproductive health 6) cognitive health; 7) general physical health.

## Results

### Literature search

A total of 702 titles and abstracts were reviewed, and 47 articles were identified for a full-text review. 38 qualitative studies were excluded (see Fig 1 for the exact reasons for exclusion). We additionally included 12 records identified manually from reference lists search, resulting in 21 articles included in the final analysis (Fig 1, Table 1). The 21 articles considered the results of 28 analyses.

**Table 1:** Overview of the empirical evidence on the associations between facial features and health outcomes.

Category of health predictors	Health measurements	Facial features	Approach to facial features	N and age	Study (first author, year)
Cardiovascular health	percentage body fat, body mass index, blood pressure	shape variation	measured 138 landmarks (GMM) perceived	135♂ 137♀ Age 18–32 y	Stephen, 2017
	body mass index	sexual dimorphism	perceived	96 ♀ M = 21.42, SD = 3.02	Han, 2016
	diabetes hypertension	FA	measured lateral view: 4 landmarks, 19 semi-landmarks, frontal view: 10 landmarks, 12 semi-landmarks	88♂ 121♀ M = 71 (60–95 y),	Nunes, 2018
	resting blood pressure, lung function, vertical jump, grip strength, sit and reach, blood lipid cholesterol, and maximal oxygen uptake	FA	measured Facial Symmetry Index Euclidean distance matrix analysis (EDMA)	21♂ M = 21.3, SD = 2.37 25♀ M = 19.5, SD = 3.44	Tomkinson, 2000
	blood pressure, history of diabetes, cardiovascular or vascular diseases	FA	measured 15 landmarks	95♂ 121♀ M = 83	Penke, 2009
Immunocompetence	overall bacterial immunity	FA averageness sexual dimorphism	perceived	101♂ M = 20.8, SD = 3.6 80♀ M = 21.9, SD = 4.6	Foo, 2017
	antibodies rise after Hepatitis B vaccine	masculinity	perceived	74 ♂ M age = 23.0, SD = 3.9	Rantala, 2013
	MHC Heterozygosity	FA averageness sexual dimorphism	perceived	80♂ M age = 20.34, SD = 3.26 80♀ M age = 19.64, SD = 2.82	Lie, 2008
	MHC	sexual dimorphism	measured 3D, mesh of 7150 points and quasi-landmarks	1 233 ♂♀ Age 18–30 y	Zaidi, 2019
Oxidative stress	urinary 8-OHdG, Cu-Zn SOD, TBARS	FA averageness health	measured (GMM) perceived	97 ♀ M = 65.5, SD = 8.89	Marcinkowska, 2020a
	urinary 8-OHdG, MDA	FA	measured landmarks of 10 bilateral features, measured with calipers	98 ♂ M = 20.1, SD = 2.9	Gangestad, 2010
	urinary 8-OHdG, 8-isoprostane	FA averageness sexual dimorphism	perceived	101♂ M = 20.8, SD = 3.6 80♀ M = 21.9, SD = 4.6	Foo, 2017
Cortisol level	morning salivary cortisol level stress-test cortisol	FA	measured 6 horizontal measures	100 ♂ M = 21.44, SD = 2.44	Borráz-León, 2017
	morning salivary cortisol	FA	measured landmarks of 10 bilateral features, measured with calipers	98♂ M = 20.1, SD = 2.9	Gangestad, 2010
	repeated measurements of salivary cortisol	sexual dimorphism	perceived	96 ♀ M = 21.42, SD = 3.02	Han, 2016
Reproductive health	semen quality	averageness sexual dimorphism FA	perceived	86-91♂ M = 20.8, SD = 3.6	Foo, 2017
	blood testosterone level	masculinity	perceived	69 ♂ M = 23.0, SD = 3.9	Rantala, 2013
	conception probability	averageness sexual dimorphism FA	measured 124 landmarks	75♀ M = 28.8, SD = 4.6	Marcinkowska, 2020b
	conception probability sex hormone levels	sexual dimorphism	perceived	88 ♀ Age 18–36 y	Marcinkowska, 2021
Cognitive health	somatization, obsessive–compulsive, interpersonal sensitivity, depression, anxiety, hostility, phobic anxiety, paranoid ideation, psychoticism, and the general psychopathology index	FA	measured 39 landmarks	168♂ M = 22.77, SD = 4.56 190♀ M = 21.21, SD = 3.28	Borráz-León, 2021
	cognitive decline (standard perceived tests and reaction time measures)	FA	measured 15 landmarks	95♂ 121♀ M = 83	Penke, 2009
	Autism-spectrum Quotient (AQ)	sexual dimorphism	measured 3D, 21 landmarks	107♂ 101♀ M = 22.81, SD = 0.63	Gilani, 2015
	Autism-spectrum Quotient (AQ)	sexual dimorphism	perceived	135 ♀ 90♂ M = 21.45, SD = 5.04	Scott,2015
General health	mortality risk	FA	perceived	133♂ 159♀ M = 83.3, SD = 0.5	Dykiert, 2012
	Physical Fitness Index	FA	measured 15 landmarks	95♂ 121♀ M = 83	Penke, 2009
	antibiotics use, respiratory infections, stomach/intestinal infections (duration, number of infections)	FA sexual dimorphism	measured 5 traits	203♂ M = 21.06, SD = 3.55 203♀ M = 19.95, SD = 3.24	Thornhill & Gangestad 2006
	antibiotic use, number of infections, antibiotic use, flu days	sexual dimorphism	perceived	105 ♀ M = 18.5 (18–20 y)	Gray & Boothroyd, 2012
	frequency and timing of infections, immunoglobulin A	sexual dimorphism averageness	measured 31 landmarks	590 ♀ M = 21.48, SD = 3.24	Cai, 2019

FA – facial fluctuating asymmetry, GMM – geometric morphometrics method, Cu-Zn SOD – copper/zinc superoxide dismutase, MDA – malondialdehyde, MHC – major histocompatibility complex, TBARS – thiobarbituric acid reactive substances, 8-OHdG – 8-hydroxy-deoxyguanosine, M – mean, SD – standard deviation, N – number of participants.

### Articles characteristic

The earliest publication identified was published in 2000 by Tomkinson et al. [5]. The latest publications included in this review were Borráz-León et al. [46] and Marcinkowska et al. [47], both published in 2021. More than half of the studies (13/21) were published since 2015, with the vast majority published since 2010 (17/21; Table 1). Most of the included articles evaluated facial sexual dimorphism, particularly femininity or masculinity (16/21), and facial symmetry or fluctuating asymmetry (17/21), while only a few addressed facial averageness (7/21).

### Facial features and various aspects of health

#### 1. Cardiovascular health.

Stephen et al. [18] measured facial shape variation (expressed as a combination of facial symmetry, averageness, and sexual dimorphism) and its relation to cardiometabolic health. They employed geometric morphometric method [48] of facial landmark data to forecast the presence of risk factors for cardiovascular disease – Body Mass Index (BMI, 32% of variance explained by facial shape, effect size R^2^ = 0.319, F_27,240_ = 5.004, p < 0.001), percentage of body fat (21% explained, effect size R^2^ = 0.210, F_28,166_ = 3.824, p < 0.001), and blood pressure (21% explained, effect size R^2^ = 0.213, F_27,240_ = 2.650, p < 0.001). Although the sample size of the study is relatively small, the quality is enhanced by the enrollment of participants of varying ethnical backgrounds. Importantly, the facial stimuli of each participant were judged by a peer of the same ethnicity. In a study by Han et al. [49], although the relationship between health and facial shape was not a primary research question, BMI was found to be negatively related to facial femininity (r = –0.32, p = 0.002). The study was based on university participants, photographs were taken solely of White participants, and 73% of raters identified as White. Nunes et al. [50] used a geometric morphometrics approach to identify facial shape traits associated with the presence of diabetes, hypertension, or both conditions. The standardized effect sizes (D2 Mahalanobis distances) ranged between 1.78–6.10; all p-values were <0.02, meaning that the facial shape was different between all health groups (with the exception of the comparison between men with hypertension and men with both conditions). The largest facial morphological disparity was observed between individuals without the two conditions and those with diabetes. On the contrary, for most studies, this study was based on elderly individuals, which expands the possibilities for testing whether facial features are a biomarker of health throughout the lifetime. Additionally, facial shape measures were based on both frontal and lateral photographs, which can possibly increase the quality of the measurement. Somewhat incongruently with previous studies, Tomkinson et al. [5] reported no significant relationship between health-related physiological parameters (resting blood pressure, lung function, vertical jump, grip strength, sit and reach, blood lipid cholesterol and maximal oxygen uptake using cycle ergometry) and facial asymmetry (correlation coefficients < |0.41|, all ps > 0.05). Interestingly, correlations between asymmetry of individual traits depending on the used method or trait were negligible (statistics not provided by authors), which warrants caution when using any single bilateral trait as a biomarker. Similarly, Penke et al. [51] did not report significant relationships between horizontal fluctuating asymmetry (HFA) and comprehensive fluctuating asymmetry (CFA) indices and the physical fitness factors, i.e., blood pressure, history of diabetes, cardiovascular or vascular diseases (rs < |0.11|, ps > 0.30). As in Nunes et al. [50], the study was based on elderly participants, which on one hand might provide a better depiction of the overall life health. On the other hand, elderly participants had more post-natal life exposure, and the prenatal environment might have relatively smaller effect on the current state of their bodies. In fact, the measured asymmetry of the elderly sample had a significantly higher mean [t_(364)_ =9.68, p < 0.001, d = 1.01] and increased variance [F_(1,364)_=12.39, p < 0.001] than the younger comparison sample. Nevertheless, two studies based on elderly participants and cardiovascular health show incongruent results. The difference might stem from the fact that Nunes et al. [50] based their analyses on a diagnosis of hypertension or diabetes (dichotomized variable), while Penke et al. [51] used raw measurements describing cardio-metabolic health. Additionally, Penke et al. [51] based their analyses on a cohort prospective study, which warrants a greater representativeness of the measured sample (however, fitness traits are reported as a single time point measurement, not a repeated one).

#### 2. Immunocompetence.

Foo et al. [2] indicated weak associations between perceptual facial features and various aspects of immune function. Multiple regression models showed that bacterial immunity was not related to facial appearance predictors (ps = 0.20–0.66, rs = |0.00| - |0.15|) in either men or women. The study was based on 181 White students. Gathering data from participants was standardised for both circadian rhythm and menstrual cycle for women. Overall, the study employed rigid protocols and investigated multiple aspects of health. Going further, Rantala et al. [52] reported an association between hepatitis B antibody response and facial features. They found that following a hepatitis B vaccination protocol, there was a positive association between men’s immune reaction and facial masculinity (effect size: r = 0.47, p < 0.001) rated by female participants. This is the only published study analysing immune reactivity and its correlation with facial features. Measuring vaccine reactivity mimics a reaction to infection. This provides an additional insight into measuring the immune health of an individual. While measuring solely young, healthy participants, it is possible that null results would stem from the lack of immune variation. On the other hand, Lie et al. [37] did not find a significant relationship between facial symmetry or sexual dimorphism judged by opposite-sex raters and an indirect measure of innate immunity diversity at the major histocompatibility complex, MHC (rs = |0.04|-|0.19|). However, the study revealed that facial averageness was associated positively with overall MHC heterozygosity in men (r = 0.42; p < 0.001). The same research question was posed by Zaidi et al. [53]. Based on a large sample (N = 1233) and 3D facial measures, they did not find a significant relationship between MHC heterozygosity and facial sexual dimorphism (tested by linear model, t = 0.586, p = 0.558, and t=−0.038, p = 0.970, respectively, effect sizes not reported). The study included several confounding factors: overall body size, age, weight, genome-wide heterozygosity, and population structure. The sample size was large, points on the facial 3D representation was numerous, and many confounding factors were included in the model. We believe this study constitutes solid evidence for no relationship between facial sexual dimorphism in both men and women and MHC-heterozygosity. Also, authors added a comparison of different measurements of sexual dimorphism, which yielded highly statistically significant, positive results (Electronic Supplementary Material, r > |0.818|, p values not provided).

It is possible that the incongruency between positive correlation between facial masculinity and immunocompetence found in Rantala et al. and lack of such results in Zaidi et al. [53] and Lie et al. [37] stems from the fact that latter two measured MHC region, that is a genetic factor inherited from parents – unrelated with developmental conditions. On the contrary, immune reactivity can be shaped by multiple external factors – it will show greater dependency on pre- and post-natal conditions. Rantala et al. [52] did not, however, measure either symmetry or averageness, which are putatively better biomarkers of prenatal environment than masculinity (shaped mainly during pubertal time).

#### 3. Oxidative stress level.

Oxidative stress (OS), among other factors, contributes to the development of diabetes, obesity, and diabetes-related microvascular diseases, atherosclerosis, and cardiovascular diseases [54,55] and is frequently used as a biomarker of the condition of an organism, its health, and the pace of ageing. Marcinkowska et al. [23] showed mixed results, including positive, negative, and null relationships between OS and facial features in a sample of postmenopausal women. In this study a multivariate regression analysis was used to investigate the relationship between OS levels [measured by DNA damage and 3 levels of biomarkers, namely 8-hydroxy-20-deoxyguanosine (8-OHdG), copper/zinc superoxide dismutase (Cu-Zn SOD), thiobarbituric acid reactive substances (TBARS) levels] and facial morphology features calculated using PsychoMorph Program [56]. Analyses were based on measured and perceptual approaches to facial features. Interestingly, the first analysis with perceived facial features showed that faces of women with high OS were chosen as less symmetrical (F = 4.370, p = 0.037), but healthier (F = 84.39, p < 0.001), than faces of women with low OS. The second analysis, where the Geometric Morphometric Modelling was used to calculate the facial features, did not confirm any statistically significant relationship between OS biomarkers and facial morphology (all ps > 0.449). The model controlled for age and BMI. In the third analysis negative, albeit weak, correlations between both measured facial symmetry (b = −0.096, 95%CI [−0.18, −0.01], R^2^ = 0.042, p = 0.025) and measured averageness (b = −0.140, 95%CI [−0.25, −0.03], R^2^ = 0.050, p = 0.016) and 8-OHdG were observed. Moreover, Cu–Zn SOD, was negatively correlated with averageness (b = −0.054, 95%CI [−0.10, −0.01], R^2^ = 0.038, p = 0.032), but not symmetry (p = 0.123). No statistically significant associations with either averageness or symmetry and TBARS were detected [23]. Age was not a significant covariate in the analyses, hence, it was excluded from the final model. The study included a comprehensive measurement of the oxidative stress, namely 3 separate biomarkers, and employed both measurement of the facial shape and judgment of it. Additionally, the study was based on a post-menopausal sample of rural women, which is an underrepresented group in studies of human health. Confounding variables other than age were also included (i.e., BMI).

Gangestad et al. [57] also explored 8-OHdG as a marker of OS levels and additionally malondialdehyde (MDA), which is a marker of lipid oxidative damage. Measured FA significantly and positively predicted levels of urinary OS biomarkers (partial r = 0.26, p = 0.021) for aggregated OS biomarkers and 8-OHdG (partial r = 0.24, p < 0.05). The second OS biomarker (MDA) was not related to the FA (r = 0.16, no p value reported). The model controlled for toxin exposure, smoking, and their interaction. Moreover, the study found no evidence that either cortisol or testosterone mediates the association between FA and OS levels (no numeric results reported). Although the analysis was based solely on young men (students), it did control for some important confounders known to influence OS (smoking and toxin exposure). On the contrary, Foo et al. [2] showed no significant relationship between facial features and OS measures in women and men (all ps < 0.0.19). The study was based on a sample of university students; two measures of OS were included: urinary 8-OHdG and 8-isoprostane. The analyses controlled for age and various lifestyle variables.

#### 4. Cortisol level.

Cortisol is a steroid hormone that performs various roles in the human body, including managing the stress response, regulating metabolism, immune function, and inflammatory response [58,59], and is frequently used as a biomarker of chronic stress exposure and health deterioration resulting from it. Analyses based on facial measurements in Borráz-León et al. [60] showed no significant associations between FA and morning salivary cortisol levels (p > 0.05). However, after a stress test, symmetrical men (with lower FA) showed an increase in cortisol levels, whereas asymmetrical men (with higher FA) exhibited a decrease in cortisol levels (b = 0.788, Wald = 61.421, p < 0.001). The analyses were based on university students and employed rather general facial shape measurements, based on 6 horizontal measures. The advantage of the study is measuring a hormonal response to stress, not solely morning hormonal levels. Models controlled for perceived stress and competitiveness scores. Another study by Gangestad et al. [57] did not detect a significant relationship between cortisol level and FA (p = 0.755). Results come from the same article as results for oxidative stress and FA analyses. Likewise, Han et al. [49] did not observe significant correlations between cortisol levels (on a single session or averaged over 5 test sessions) and ratings of facial femininity in women (r = 0.09, p = 0.37 and r = −0.08, p = 0.43 respectively). The study was based solely on university students, and cortisol was measured in two different ways (averaged and on a single session). The authors report exclusion criteria (recent breastfeeding, contraception, or pregnancy), but do not report on any confounding variables. Summarizing, the sole study that showed a significant relationship of cortisol with facial shape (Borráz-León et al. [60]) measured a stress response, not a hormonal baseline.

#### 5. Reproductive health.

Foo et al.[2] measured the relation between facial features and semen quality in men. They indicated that the linearity of sperm movement was positively predicted by facial masculinity (effect size: r = 0.29, p = 0.01). Furthermore, sperm concentration and percentage of motile sperm were negatively predicted by facial averageness (r = −0.21, p = 0.04) and positively by facial masculinity rated by opposite-sex judges (r = 0.23, p = 0.03). Facial symmetry was not related to either of the sperm quality measures (ps > 0.16). Although semen quality is not a direct measure of sexual fitness, the study provides complex information about semen biological quality, which is critical for male fertility. Interestingly, symmetry was unrelated to the reproductive measures, and masculinity seemed to have the strongest effect. The study controlled for age and various lifestyle variables. Rantala et al. [52] showed that facial masculinity was significantly correlated with circulating testosterone levels (r = 0.38, p = 0.001) in men. Authors do not report on confounding variables. Nevertheless, the sample was quite homogeneous – White students of one of the Latvian Universities. On the other hand, a study of Marcinkowska et al. [61] tested facial shape correlates of fertility in women. They found no evidence for a change in facial asymmetry, averageness, or sexual dimorphism between three different points in the menstrual cycle, which vary in conception probability, and levels of female typical sex hormones, namely estradiol and progesterone (Fs ≤ 0.78, partial η2s≤0.01, ps ≥ 0.542). The results remained the same when the analysis was conducted solely on women who obtained a positive ovulation test result (N = 35). Similarly, as with semen quality, ovulation is not a direct measure of reproductive success, but it does provide information on reproductive potential and reproductive health. The study showed that expected cyclical fluctuations in conception probability are unrelated to facial shape. The study sample was not large, but it accounted for between women variation in ovulation timing – the fertile window gauging was based on an actual LH peak, not solely on a self-report or counting days method. In another study, Marcinkowska et al. [47], using a complex methodological approach, found limited evidence for the relation between perceived femininity, conception probability, and sex hormones. The study employed a comparison of results from 3-Alternative Forced Choice (3-AFC) and Likert Scale judgements, inclusion of varying visual stimuli, and was based on a cross-cultural sample of raters. The reproductive health biomarkers included estradiol, progesterone, estradiol/progesterone ratio, and conception probability based on an ovulation test. For Likert Scale judgements, estradiol was significantly related to facial femininity (estimate = 0.09, SE = 0.03, p = 0.011), all other relationships were insignificant (estimates < |0.11|, ps > 0.156). For 3-AFC, the E/P ratio was significantly related to femininity (estimate = 0.17, SE = 0.09, p = 0.046), all other relationships were insignificant (estimates<0.23, ps > 0.086). Even more importantly, the two statistically significant effects differed depending on the rating method – forced choice vs. Likert Scale. Strikingly, associations between the two rating approaches were not strong. The only association (out of 5) that was statistically significant was just below the threshold of significance(p = 0.046) and would not survive the multiple comparisons correction. This result provides methodological evidence for the complexity of the relationship between facial judgement and facial features, and the importance of the type of methodology employed.

In a meta-analysis, Lidborg et al. [62] analysed studies relating facial masculinity to reproductive measures, i.e., number of children, grandchildren, age at birth of the first child, and number of offspring surviving childhood. Although reproductive success is not unequivocal with reproductive health, we can safely assume the two are highly correlated. The authors report that the only significant predictor of the reproductive domain was body masculinity (r = 0.143, 95%CI: 0.076, 0.212, p < 0.001), with the effects of facial masculinity on reproduction not reaching the threshold of statistical significance (r = 0.099, 95%CI: −0.012; 0.211, p = 0.081). It is worth mentioning that authors also accounted in the analyses for high and low fertility between samples, divided reproduction into a couple of domains, and added subgroup and moderator analyses, all models not showing a significant relationship between reproduction and facial masculinity (ps > 0.067). Hence, the meta-analysis provides solid proof for the lack of grounds to interpret facial sexual dimorphism as a proxy for higher *reproductive quality* of an individual.

#### 6. Cognitive health.

Borráz-León et al. [46] showed no correlations (in a mixed group of both men and women) between facial symmetry and “minor mental ailments” Somatization, Obsessive–Compulsive, Interpersonal Sensitivity, Depression, Anxiety, Hostility, Phobic Anxiety, Paranoid Ideation, Psychoticism, and the General Psychopathology Index, measured via Symptom Checklist-90-Revised (SCL-90-R), (rs < 0.10, ps > 0.05). The study was based on university students and accounted for confounding variables, i.e., age, sex, and BMI. Penke et al. [51] showed that facial asymmetry indices (namely horizontal fluctuating asymmetry, HFA; and comprehensive fluctuating asymmetry index, CFA) at age 83 were unrelated to measured intelligence assessed at age 11, 79, or 83 (all ps > 0.05). Facial asymmetry at age 83 was also not related to change in cognitive abilities from age 11 to age 79 years (p > 0.05). However, cognitive decline between age 79 and 83 in men was significantly, negatively related to all symmetry indices (rs=−0.24 to −0.35, 0.001 < ps < 0.05); men with lower FA at age 83 had experienced less cognitive decline in the preceding 4 years, and showed faster reaction times (but not on all types of tests, rs = .05 –.30, 0.001 < ps < 0.05). This effect was not replicated in women (all ps > 0.05). The study was based on elderly participants recruited for a prospective cohort study, which can increase the generalizability of the obtained results. Also importantly, the authors run repeated measure models, and can gauge the actual change, or lack thereof, in cogitative abilities. Gilani et al. [63] showed that men and women with high levels of autistic-like traits present less prominent facial sexual dimorphism than individuals with low levels of autistic-like traits (for 4 out of 6 facial measurements for men R^2^ > |0.13|, ps < 0.05; and 3 out of 6 for women R^2^ < |0.16| ps < 0.003). One of the measurements (nasal bridge length) showed an opposite pattern in women – shorter average nasal bridge length (more feminine) was observed in a group of higher autism scores (R^2^ = 0.21, p = 0.001). The analysis was conducted on a population-based, longitudinal pregnancy cohort study. Contrary to Penke et al. [51], the analyses included only single-time-point measures of the outcome variable. The study sample is more representative than the predominantly university-based sample.

Scott et al. [64] found that morphed photographs (averaged facial representation based on real photographs) of men who scored higher in Autism Spectrum Quotient (AQ) were judged as more masculine, across three different experiments, varying slightly in methodological approach. In study 1, observers selected high-AQ male faces as more masculine 69% of the time, a rate significantly higher than that expected by chance (t(37)=6.07, p < 0.0001, R^2^ = .49). In Study 2, observers picked the high-AQ male face as more masculine 69% of the time, a level significantly higher than chance, t(47)=6.61, p < 0.00001, R^2^ = .48. The results were similar (direction and strength of the effect) when presenting the judges with composites based on either 6 or 15 real face photographs. Study 3 showed similar results for all 5 subscales of the Autism Spectrum (R^2^s>0.24, ps < 0.001). In all studies, there was no effect detected for female faces. The studies controlled for ethnicity, age, and facial hair by excluding participants who did not fit the inclusion criteria. Overall, the effect found in this series of studies seems robust, and effect sizes are comparable between studies. The facial stimuli on which the autism-masculinity relationship was found were based on a sample of students, which could be a limitation, and could lead to the discrepancy between these and Gilani et al. [63] results (who found a significant relationship also in women).

#### 7. General health.

Dykiert et al. [65] indicated that rated facial symmetry was not associated with the risk of mortality in a group of elderly participants with an average age of 83 years (p = 0.55 for the whole sample; p = 0.32 for men; p = 0.58 for women). Participants were selected from a birth cohort prospective study. The strength of the study is the fact that the participants were measured over 7 years. Models included covariates: sex, chronological age, systolic, diastolic blood pressure, grip strength, Raven SPM score, and Mini-Mental State Examination result. Also, Penke et al. [51] did not report significant relationships between any of the two measured symmetry indices (HFA, CFA) and the physical fitness factors for either sex (rs < |0.11|, ps > 0.30). That study was based on elderly participants recruited from a cohort study. Physical fitness factors were based on time of walking 6 meters, grip strength, and forced expiratory volume from the lungs. Confounding variables included: history of diabetes, cardiovascular disease, high blood pressure, and other vascular diseases. It is worth mentioning that the above-mentioned studies both show null results, while being based on dependent variables that together describe very well the overall physical state: fitness factors and mortality risk, which is a direct consequence of them. Thornhill & Gangestad [66] demonstrated that facial masculinity interacted with sex, and predicted the number of reported occurrences of antibiotic use (measure of effective immunocompetence) and respiratory infections (number of infections estimate = −0.19, p = 0.001, days of infection estimate = −0.17, p = 0.002, effect stronger for males than for females). No such association was observed for stomach/intestinal infections (p > 0.05). Moreover, FA was not related to the total number of infections (p = 0.139), and only marginally predicted the total days infected (p = 0.07). On the other hand, FA was positively associated with days (estimate = 0.14, p = 0.011) and number (estimate = 0.11, p = 0.03) of respiratory infections, but not with stomach infections. It is worth mentioning that the association between FA and the number of respiratory infections was only marginally significant when controlled for potential confounders (age, height, weight, and socio-economic status). No effect was observed when FA and intestinal ailments were analysed (p > 0.05). Facial asymmetry was marginally related to the number of times that antibiotics were used (p = 0.057) for both sexes combined. In a similar study, Gray and Boothroyd [30] examined whether perceived femininity was related to infections and antibiotic use of young adult women. They found a trend for facial femininity to correlate negatively with the reported number of colds in the preceding twelve months (r=−2.15, p < .05), with the frequency of antibiotic use in the last three years (r= − 0.272, p = 0.007) and the last twelve months (r= −0.211, p = 0.039), and with days of flu (r=−.265, p ≤ .05). No such effects were found for stomach illnesses neither for number of colds in last 3 years, number of illnesses, colds, flu, or days of antibiotic use in the last 8 weeks (all ps ≥ 0.324). The study shows a similar trend in almost all measured health variables, but not in the short term (past 8 weeks) – it may be due to the lower amount of illness among young adults in the 2 months before completion of the study. All participants were university students from two adjacent academic years. The authors controlled for rated makeup and mood of the participants. Another study based on female university students (Cai et al. [9]) report no significant correlations between most aspects of facial appearance (measured femininity and averageness) and 1) number of infections in last year, 2) timing of the last infection, 3) level of immunoglobulin A, and 4) upper respiratory symptoms in last week (last all absolute rhos < .123, all ps > .070, for the last dependent variable the p value was below 0.05 threshold, but it did not survive to correction for multiple comparisons). Facial sexual dimorphism was measured in two distinct ways. The results remained the same after controlling for participants’ age. Although the study is based on a homogeneous group (students, close in age, 98% White), the sample is large, which increases the chances of detecting the existing relationships. Also, authors point out that various health measures were only weakly intercorrelated, which shows the importance of analysing multiple health measures in studies testing relationships between health and facial shape. Confirming this claim, two studies investigating illness number separately for respiratory and intestinal infections did not find a statistically significant relationship between sexual dimorphism and intestinal infections, but only with respiratory ones (i.e., cold, flu). Then, in one study, only the most recent infectious symptoms trended to be related to facial shape (however, due to multiple comparisons, the statistical significance was lost, Cai et al. [9]), and in others, only the long-term health measures (Gray & Boothroyd [30]).

## Discussion

This scoping review provides an evaluation of literature focused on the relationship between the most frequently analysed facial features (asymmetry, averageness, and sexual dimorphism) and various measures of health, including cardiovascular, reproductive, and overall health, oxidative stress level, and immune function. Across 21 articles included in the review, sample sizes vary from small to large (46–1233 participants), including cohort studies with repeated measures of health. No studies included repeated measures of facial shape. Most of the studies focused on university students, with 4 studies including only elderly participants, and three further studies recruiting from the general population in the reproductive age span (age range 18–36). The reported effect sizes were frequently negligible or weak to moderate.

The study of symmetry in the context of human sexual selection has its roots in research from biology and behavioural ecology. Initial excitement surrounding the idea that females might prefer males with more symmetrical sexual ornaments led biologists to scrutinize the reliability of these findings across species and traits. A key question emerged regarding the extent to which fluctuating asymmetry (FA) serves as an indicator of underlying developmental instability. Through modelling, Houle [67] estimated that the correlation between FA in a single trait and developmental instability is relatively weak. Building on this, Van Dongen [68] and Whitlock and Westerfield [69] developed methods to assess this relationship. Empirical evidence from Gangestad and Thornhill [70] further suggested that, across taxa, the association between FA in a single trait and developmental instability is generally low (typically with effect sizes of around 0.2). While composite FA measures, which aggregate multiple traits, may offer improved validity in evaluating developmental instability, assuming it affects the organism as a whole, combining only a small number of traits results in a measure of limited accuracy.

Our scoping review expands on a meta-analysis published in 2011 (search of literature conducted in 2009 [15]), which examined the relationship between FA and various health indices in humans, and found that body FA, but not facial FA assessments, predicted health outcomes. The effect sizes for studies including health measures (N = 3) ranged between 0.09 and 0.13, [71]) provided evidence for a genuine association and produced effect size estimates closely aligned with those reported by Van Dongen and Gangestad [15]. Hence, it is possible that facial asymmetry alone (not combined with other asymmetry measures) does not provide a sufficient input for gauging prenatal environments of an individual.

There are potential implications for the idea of facial features signalling. The FA of a single trait is unlikely to provide reliable information to receivers about the key underlying factor – the quality of prenatal environment/developmental stability. If facial symmetry is regarded as the FA of a single trait, it seems improbable that it would offer choosers a meaningful insight into an individual’s overall “quality”. In practice, however, facial FA is often measured by aggregating multiple asymmetries. Yet, this does not necessarily mean these asymmetries are independent. Multiple facial asymmetries (such as differences in the width of the left and right sides of the face) may largely stem from the growth of the same set of developmental structures. Gangestad and Thornhill [72] explicitly examined facial FA in this context, noting that in their sample, facial FA and bodily FA were uncorrelated (r = .087 for men and r = .014 for women). More broadly, FA in individual traits tends to show only weak correlations with developmental instability (Gangestad & Thornhill [70]), making it unsurprising that facial FA does not strongly align with a composite measure of bodily FA, though, at the population level, a slight positive correlation would be expected. If facial FA primarily captures what is functionally equivalent to a single trait’s asymmetry, it cannot be expected to be preferred specifically because it signals quality.

Other than the actual lack of relation between facial features and health in published studies, there can be multiple reasons for the discrepancy in the results. The articles included in the review included adult populations from Europe, Asia, Africa, and the Middle East. Participants in the study ranged in age from 18 to 83. Twelve studies involved male as well as female participants, 3 focused only on male participants, and 6 only on females. In most of the studies, the participants were predominantly White, with the exception of one (Stephen et al. [73]), where the group included individuals from Asian, Black, and White ethnicities. There is a need to expand research to include underrepresented ethnic groups.

The pronounced variation on the sampling level could have led to varying results and effect sizes of the reported significant effects. Additionally, the differences between results could stem from different methods of measuring facial features, and also from a great array of employed measures of health. Measuring the facial features differed in protocols and the number of landmarks. The perceptual approach (judgements of the facial features) in most, but not all, publications relied on other-sex raters. Also, most studies did not report between and within-rater agreements.

### The significance of attractiveness and facial adiposity

When considering face as a biomarker of health, two more aspects seem to be closely related, facial attractiveness and adiposity. As facial attractiveness refers to other people’s perception, it cannot be directly measured (as with facial symmetry or averageness). If face was a biomarker of health, then facial appearance could serve as an honest signal manifesting mate quality to potential mates. Taking the evolutionary lens, individuals who would find “healthy faces” attractive would then be able to obtain better genes or/and improved fitness for their children. Published studies present mixed results for the relationship between the three facial characteristics of interest (sexual dimorphism, averageness, and symmetry) and attractiveness. Although theories of sexual signalling predict that attractive appearance would be positively related to actual health [2,74,75], not all studies found such a connection.

Foo et al. [2] found that neither symmetry nor averageness significantly predicted perceived attractiveness in females. On the other hand, Rantala et al. [52] showed that antibody response was significantly correlated with facial attractiveness (r = 0.43, p < 0.001). Similarly, Lie et al. [37] showed that MHC heterozygosity positively predicted male attractiveness [37]. Gangestad et al. [57] found a modest negative correlation between male physical attractiveness and Oxidative Stress (OS) biomarkers levels, but Foo et al. failed to find such a relationship [2]. Interestingly, Marcinkowska et al. reported that faces of women with high OS were perceived as more attractive [23]. There are also mixed results on the relationship between attractiveness and plasma cortisol level [59,76]. In the overall health domain, Thornhill & Gangestad presented no associations between facial attractiveness and total infections and antibiotic use [66]. No correlations were found between minor ailments in mental health outcomes and other-perceived attractiveness, however, self-perceived attractiveness was a significant predictor of health among both men and women [46]. Attractiveness also did not significantly predict mortality [65]. These strongly mixed findings suggest that even if facial symmetry and averageness would indicate health, the perception of attractiveness based on these cues may not be as universally consistent as previously expected [2,61]. Importantly, if the components of attractiveness were symmetry, averageness, and sexual dimorphism, then evidence for the interpretation of attractiveness as an adaptive signal of health is not as strong as assumed.

Also, it is possible that individuals who strongly prioritize symmetry (find more symmetric faces more attractive) in mate selection may not do so because symmetry reliably indicates developmental health or genetic fitness. Enquist and Arak [77] and Johnstone [78] have proposed that symmetry preferences might instead arise from the exploitation of sensory biases. Further theoretical modelling and empirical research are necessary to clarify this issue (Gangestad & Thornhill [72]; in Polak et al. [16] ed.). Supporting this approach, a somewhat related study (although not measuring health directly, but solely facial attractiveness), confirmed that neural “sparseness” (“the sparseness of neurons in the visual cortex required to represent a given face image thought to predict attractiveness because they index image-coding efficiency” Renoult et al. [79]) is a better predictor of facial attractiveness than BMI, sexual dimorphism, averageness and symmetry (Holzleitner et al. [80]).

Another theoretical concept that is closely related to facial appearance and health is adiposity. Foo et al. showed that men’s perceived health was negatively predicted by facial adiposity [2]. A study by Coetzee et al. indicated that facial adiposity can act as a cue to health in young adult participants [81]. Research also showed a negative correlation between adiposity and immune response in men [52]. Interestingly, women’s adiposity did not correlate with immune responsiveness [76]. The review of de Jager et al. [82] presented 3 studies that investigated the relationship between facial adiposity and mental health. One of these three articles found that rated facial adiposity was negatively correlated with a psychological condition in women [82,83]. Due to the possible relationship between adiposity and health, and adiposity and facial appearance, future studies could include a measurement of it as a confounding factor. Nevertheless, in the current study, we aimed to present studies that were investigating symmetry, averageness, and sexual dimorphism as possible markers of early-life developmental stability, and thus potential predictors of health in adult life. As facial adiposity is strongly related to post-natal life (including the environment and life-style choices [84,85]) and therefore it is a highly fluctuating parameter across the lifespan, we did not include studies exploring facial adiposity in our manuscript. However, it cannot be unnoticed that facial adiposity could serve as a marker of the current health status of an individual.

### Strengths and limitations

This scoping review can serve as a valuable resource that presents the existing evidence for the linkage between health and facial shape, offering the characteristics of the included studies, highlighting gaps, and areas requiring further research. The strength of this scoping review is the standardized literature screening process and the evaluation of the extracted data by multiple reviewers, which increases the credibility and validity of the review. The scientific transparency and open science approach (https://osf.io/dv9pu/) provides a foundation for subsequent systematic reviews or primary research to investigate the evidence on the relationship between health and facial features, employing various measurement approaches across different populations and settings.

Nevertheless, there are several limitations to our scoping review that should be noted. Firstly, the search strategy’s scope, either involving restrictive inclusion/exclusion criteria and the selected databases, may not encompass all relevant literature on the research topic, objectives, and questions. Secondly, language restrictions by focusing solely on articles published in English could omit evidence published in other languages. In addition, we have not included “grey literature,” evidence published in forms other than scientific articles.

## Conclusion

This review sheds light on the hypothesis that facial features are a biomarker of health [1]. The majority of studies to date on this topic have not confirmed this assumption, with most reporting negligible to weak effect sizes, and only a few indicating moderate effects. Some of the studies also found results opposite than expected (worse health was related to better facial judgements or measurements). It is plausible that the previously assumed interpretation of face as a biomarker of good health and beneficial developmental conditions lacks a scientific, evidence-based grounding. More studies are needed that comprehensively test the relationship between facial features and physical and cognitive health, especially in the areas where results are most incongruent, i.e., immune health, oxidative stress, and reproductive health. A meta-analytical approach would be an advisable future direction allowing for another step forward in understanding the relationship between health and facial shape.

Additionally, with more studies employing standardised approaches based on publicly available protocols, it might be possible to either rule out previous hypotheses or to add more knowledge to support their scientific grounding. Currently, we cannot conclude that there is sufficient support for the hypothesis that the human face contains valid cues to health, and that facial appearance therefore provides a reliable cue for identifying healthy and unhealthy individuals.

### Patient and public involvement

Patients and/or the public were not involved in the design, or conduct, or reporting, or dissemination plans of this research.

## Supporting information

S1 Table Preferred Reporting Items for Systematic reviews and Meta-Analyses extension for Scoping Reviews (PRISMA-ScR) Checklist. (PDF)
